# Stable Isotope Ratios in Hair and Teeth Reflect Biologic Rhythms

**DOI:** 10.1371/journal.pone.0000636

**Published:** 2007-07-25

**Authors:** Otto Appenzeller, Clifford Qualls, Franca Barbic, Raffaello Furlan, Alberto Porta

**Affiliations:** 1 New Mexico Health Enhancement and Marathon Clinics Research Foundation, Albuquerque, New Mexico, United States of America; 2 Department of Mathematics and Statistics, University of New Mexico, Albuquerque, New Mexico, United States of America; 3 Medicina Interna II, Ospedale L. Sacco, Università degli Studi di Milano, Milan, Italy; 4 Dipartimento di Tecnologie per la Salute, IRCCS Istituto Ortopedico Galeazzi, Università degli Studi di Milano, Milan, Italy; New York University College of Dentistry, United States of America

## Abstract

Biologic rhythms give insight into normal physiology and disease. They can be used as biomarkers for neuronal degenerations. We present a diverse data set to show that hair and teeth contain an extended record of biologic rhythms, and that analysis of these tissues could yield signals of neurodegenerations. We examined hair from mummified humans from South America, extinct mammals and modern animals and people, both healthy and diseased, and teeth of hominins. We also monitored heart-rate variability, a measure of a biologic rhythm, in some living subjects and analyzed it using power spectra. The samples were examined to determine variations in stable isotope ratios along the length of the hair and across growth-lines of the enamel in teeth. We found recurring circa-annual periods of slow and fast rhythms in hydrogen isotope ratios in hair and carbon and oxygen isotope ratios in teeth. The power spectra contained slow and fast frequency power, matching, in terms of normalized frequency, the spectra of heart rate variability found in our living subjects. Analysis of the power spectra of hydrogen isotope ratios in hair from a patient with neurodegeneration revealed the same spectral features seen in the patient's heart-rate variability. Our study shows that spectral analysis of stable isotope ratios in readily available tissues such as hair could become a powerful diagnostic tool when effective treatments and neuroprotective drugs for neurodegenerative diseases become available. It also suggests that similar analyses of archaeological specimens could give insight into the physiology of ancient people and animals.

## Introduction

Living matter has its own biological time: a set of rhythmic oscillations, paced by changes in gene expression in the anterior part of the hypothalamus, an area regarded as the “master time keeper”. These changes in gene expression drive a large network of intracellular clock-proteins [1]. The clock proteins, linked through feedback loops that create molecular oscillators, generate time signals that reach peripheral tissues and affect the cell cycle [2], the circadian period, and thermoregulation. The “master” time keeper and the pathways through which its signals reach peripheral tissues are part of the autonomic nervous system (ANS) [3].

These clock-like signals affect the growth of tissues including hair and teeth. Hair, for example, is renewed throughout life; it has a well-defined variability in growth, dependent on diet, age, blood flow to the skin, hormonal and metabolic status and other influences. The ANS controls many of these factors. We now show that analyses of stable isotope ratios along the length of hair and along growth lines in teeth reveal biologic rhythms and their control by the ANS.

Hydrogen isotope ratios in tissues depend on these ratios in food and water. In turn, these vary greatly from place to place. We, therefore, chose our materials for analysis from diverse geographic settings. ANS control of biologic rhythms is also diverse depending on animal size, metabolism, thermoregulatory needs and life styles. Hence our analysis included a diversity of taxa with different ANS control levels of biologic rhythms such as humans, horses and mammoth.

## Results

The annual growth rate of human hair, determined from the sinusoidal variation in hydrogen isotope ratios in our samples closely corresponded to the average reported in the literature of ∼16 cms/year [4]. For the young Italian woman 16.9 cms; the patient with pure autonomic failure (PAF) 16 cms; for South American mummies 12 cms. and for high altitude samples (4550 m.) Ladakh, 17 cms. For the horse 46 cms. and for the mammoth 31 cms. (Text S1)

Tooth growth increments visible on the surface of enamel (perikymata) are known to represent ∼9 days of growth in humans. The periodicities of perikymata growth increments for *Paranthropus robustus* was assumed to be 7 days [5]. Fifty growth lines in enamel of teeth were taken to represent yearly growth, on average, in this early hominin [6].

Using spectral indices of hydrogen isotope ratios along the length of the hairs we identified recurring annual rhythms at slow periods (SP) and fast periods (FP).

**Figure 1:**
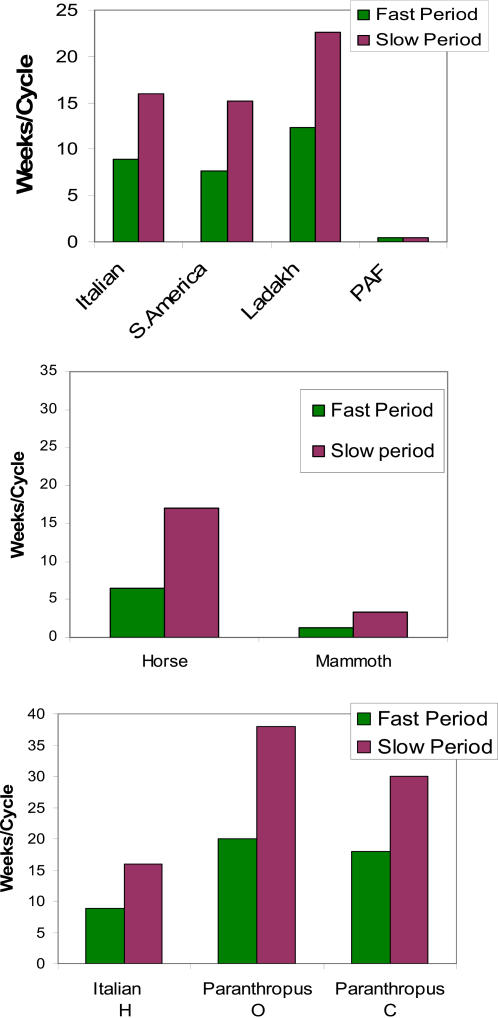
Slow and fast periods of hydrogen isotope ratios (H) from hair and oxygen (O) and carbon (C) isotope ratios from teeth of *Paranthropus robustus.* The fast periods occurred every 1.2 weeks in the mammoth. The fastest cycling in all our subjects. This is consistent with fast growth rate and the thermoregulatory function of hair, of importance to this animal living in western Siberia. The patient with PAF had neither slow nor fast periods in keeping with the absence of autonomic modulation of biologic rhythms in this neurodegenerative disease. Both, fast and slow periods, were delayed in the high altitude dwellers of Ladakh, most likely the result of life long chronic hypoxia. Teeth, unlike hair, grow only during early childhood and adolescence. And the hominin was a vegetarian. This may account for the differences in slow and fast periodicities between the hydrogen isotope ratios in hair of the Italian woman and the carbon and oxygen isotope ratios in teeth of hominins. PAF = patient suffering from pure autonomic failure.

The fast period peaks recurred at varying intervals in humans ranging from 8.9 to 12.3 weeks/cycle. In the horse and mammoth, ranging from 6.5 to 1.2 weeks/cycle respectively. In hominin teeth for oxygen isotope ratios the fast period was 20 weeks/cycle and for carbon isotope ratios 18 weeks/cycle (Fig. 1)

The slow periods (Fig 1) recurred at varying intervals in humans ranging from 16 to 22.6 weeks. However, the young Italian woman had an additional very slow period peak at 32 weeks/cycle. In the horse and mammoth the slow periods ranged from 17 to 3.25 weeks/cycle respectively. In hominin teeth for oxygen isotope ratios for the slow period peak was 38 and for carbon isotope ratios, 30 weeks/cycle. The patient with PAF had neither slow nor fast period peaks.

**Figure 2:**
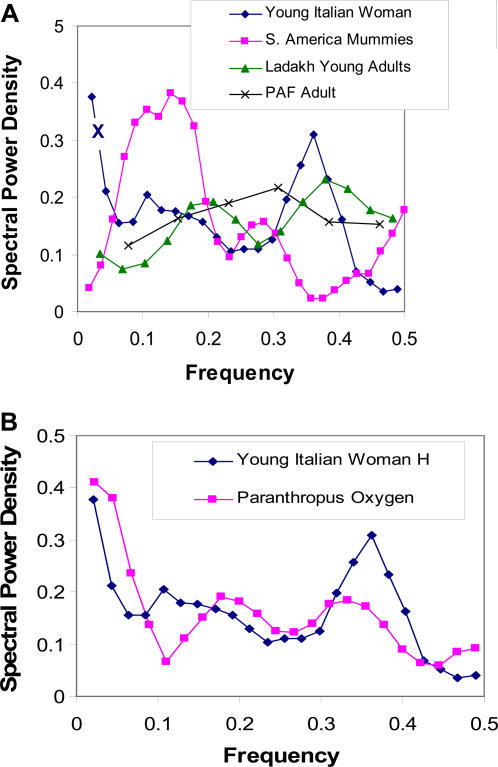
Power spectra derived from hydrogen isotope ratios in hair of humans (A) and oxygen isotope ratios in *Paranthropus robustus* teeth (B). Note that the slow and fast power peaks, in terms of normalized frequencies, are similar to those derived from power spectra of heart rate variability of the Italian subject and especially the virtual absence of frequency modulations in the patient with PAF (see Figure 3). X =  very slow frequency cycling at 32 weeks. The frequency of spectral power peaks of hydrogen isotope ratios in hair in the normal human and oxygen isotope ratios in *Paranthropus robustus* is also very similar (B) giving additional support to the influence of the ANS on biologic rhythms derived from different tissues.

The power spectral densities of the hydrogen isotope ratios obtained from hair are given in Figure 2.

**Figure 3:**
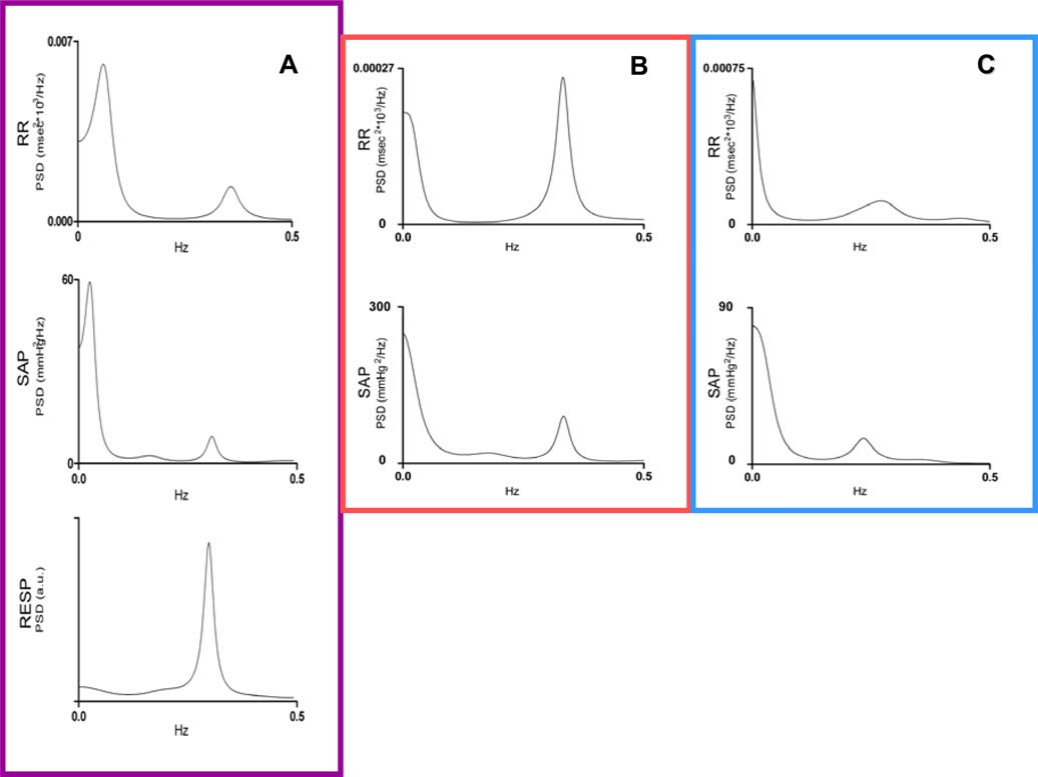
Power spectra derived from heart rate variability. A = normal young Italian woman. B =  patient with PAF recumbent. C =  same patient on upright tilt. Note low and high frequency power occur, when normalized, at similar frequencies to those obtained from hydrogen isotope ratios from these same subjects (see Fig. 2). In the patient with PAF only a high frequency peak in heart rate variability can be seen. The high frequency peak is due to respiration, as is shown in A. The low frequency peak could not be observed on head-up tilt when it normally increases in amplitude due to sympathetic activation. In this patient heart rate variability is not modulated by the ANS; this is also reflected in the spectra derived from his hydrogen isotope ratios (Fig. 2). RR = heart beat intervals; SAP = systolic arterial pressure variability; RESP = variability in respiratory frequency.

Power spectral densities of heart rate (RR), systolic blood pressure (SAP) and respiration (RESP) of the healthy Italian female and of the PAF patient are shown in Figure 3.

**Figure 4:**
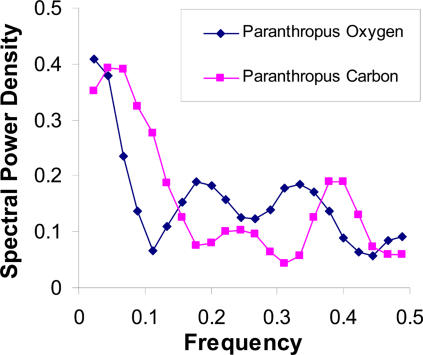
Power spectra derived from tooth enamel growth lines of carbon isotope and oxygen isotope ratios of *Paranthropus robustus,* a hominin that lived in the South African savanna ∼1.8 million years ago. The carbon isotope ratios provide a measure of the type of diet consumed and a guide to local climate at the time. The oxygen isotope ratios also shed light on diet and climate. The oxygen isotope ratio increases with surface temperature of water, evaporation and with low humidity. This in turn influences the water content in leaves from which most herbivores, including this hominin, satisfy their water requirements (raw data from on line supplement to reference 6). Note the slow and fast power peaks of the stable isotope ratios, characteristic of ANS modulation of food and water intake and the similar power spectral peaks of oxygen to those obtained from hydrogen isotopes in hair of living normal humans (see Fig. 2). Carbon isotope ratios are shifted to the right.

The power spectra densities of the carbon and oxygen isotope ratios obtained from the hominin teeth are shown in Fig 4.

## Discussion

### a) Stable isotopes

Stable isotope analysis has become a powerful, non-invasive probe for animal and human behavior. Isotopes are atoms of the same element that have the same number of protons and electrons but different numbers of neutrons; stable isotopes, unlike radioactive isotopes, do not decay to other isotopes over geologic time. Hydrogen has two stable isotopes. For hydrogen, the average terrestrial abundance ratio (^2^H∶^1^H) is−1∶6410, but the ratios vary significantly from place to place, giving rise to isotope-landscapes or “isoscapes” [7]; analysis of hydrogen isotope ratios in animal tissues can reveal movements of animals in remote locations.

Our selection of samples from around the world which extended from South America to the Himalayas, Europe and Siberia, and at high altitude as well as sea level insured that the stable isotope ratios in food and water would reflect varying isoscapes. This accounts, to some extent, for the differences in the power of the spectra derived from samples from different geographic locations (Figs. 1 & 2). This difference in stable isotope ratios is also expressed in the circa-annual frequency bands of the time series. Additionally, some part of the variations in the power spectra can be attributed to the different neuro-autonomic influences on food and water intake determined by varying life-styles and by altitude.

### b) Hair

Hair has many biologic functions including protection from the environment. It also makes significant contributions to thermoregulation, an important part of ANS function.

Throughout life each hair follicle cycles between three stages: growth, involution and rest. This cycling is under the control of molecular signals, which have been documented in mice and inferred in humans from clinical observations. Hormonal levels, aging, beta-adrenergic antagonists and many other drugs have profound effects on hair biology [4].

Hair consists of a protein-based keratin. [8]. Thus by comparing isotope ratios in a given sample with isoscapes the origin of the hair can be determined. Variations in hydrogen isotope ratios in hair from the mammoth from Western Siberia allowed the rate of hair growth and the extent of its seasonal migrations to be calculated for this extinct animal [8].

Circannual rhythms are well known in mammalian physiology. They allow animals to predict seasons by changes in endogenous cycles, in physiology and in behavior. Recently circannual regulation of hormone secretion by melatonin-regulated timer cells has opened annual pacing mechanisms to molecular analysis [9]. Our analysis of hair growth based on time series of the variations in hydrogen isotope ratios along the length of hairs confirmed the average growth rate gleaned from actual measurements.

Hair remains in the growth-stage for 2–8 years to produce long hair. Short eyebrow hairs remains in the growth phase for only 2–3 month at a time. We propose that the recurring fast period cycles we found in the human samples reflect the growth-stage of the hair. Not surprisingly the differences between fast-periods in various specimens varied with location, depending on the isotopic composition of food and water intake and autonomic modulation of life styles. We speculate that the variations in intervals from continent to continent and from prehistory to the present correlate with the nutritional state of the subjects and the altitude at which they lived. In pre-Columbian South America nutrition differed from present day norms in Italy and Ladakh and autonomic modulation of the period's life-style was also presumably different.

The slow period annual rhythms in hydrogen isotope ratios, which recur at slower intervals, align best with the rest-stage of scalp hair cycling. This stage typically lasts for 2–3 months before the hair follicle enters the growth-phase again. Since the ANS influences hair growth, less ANS modulation is expected during the rest stage. The young Italian woman's annual cycling was remarkable for two slow period rhythms at 16 and 32 weeks/cycles. The very slow, 32 week period in hydrogen isotope ratios can also be found in her heart rate variability power spectrum. This is identified in Fig. 2 by an X. Such very slow periods are not known to have physiologic correlates in autonomic control of heart rate variability. This supports our spectral method of analysis of stable isotope ratios in hair. Removing this very slow periodicity from the time series brings the annual periodicities of all ancient and modern human hair samples to comparable levels.

The patient with PAF, a neurodegeneration characterized by extensive failure of the ANS, showed no evidence of slow, or fast annual periodicities in hydrogen isotope ratios, as expected. The absence of autonomic modulation of his heart rate variability also supports our contention that the autonomic nervous system influences power spectra obtained from hydrogen isotope ratios in hair.

Anatomic and behavioral links between the ANS and hair are well established. The hair follicles have the greatest number of nerve fibers of any skin component. This neural network is continuously remodeled in step with the cycling that accompanies the three-stage biologic rhythm of hair [10]. The arrector pili muscle, closely apposed to the hair follicle, has an intimate anatomic link with the ANS; it is innervated with nerve fibers that, when activated under adrenergic influence, cause the muscle to contract and the hair to stand on end.

We now provide additional linkage using power spectral analysis of isotope ratios to show that ANS-entrained biologic rhythms are preserved in hair

### c) Power spectra using stable isotope ratios

We obtained power spectra of heart rate variability by accepted techniques [11] using electrocardiography from the normal Italian woman and the PAF patient. The spectra from the normal subject showed the expected low and high frequency peaks known to reflect the modulation of the heart rate intervals by the sympathetic and parasympathetic branches of the ANS, respectively.

PAF, a neurodegenerative disease, is characterized by widespread failure of ANS function. The power spectra of heart rate variability from this patient reflected his ANS failure. Although high frequencies were detected, these frequencies are the result of respiratory drive on heart rate. Tilting to the upright position had no effect in this patient either. Normally, the upright posture increases low frequency power in heart rate variability resulting from activation of the sympathetic autonomic drive to the heart (Fig. 3). Thus autonomic modulation of his heart rate variability was shown to be absent. Our analysis of the hydrogen isotope ratios of hair from the PAF patient revealed no slow- or fast periods either. Therefore the results from hair matched, when normalized, the spectra obtained from his heart rate variability and confirmed the patient's autonomic failure (Fig. 3) [12].

Our successful application of the method for studying the autonomic influences on heart rate variability to the power spectra constructed from hydrogen isotope ratios in hair implies that the ANS also influences the variation in these ratios, most likely by modulating food and water intake and life style.

To further support our observations on hydrogen isotope ratios in hair, we performed a complementary analysis on stable isotope ratios (^13^C/^12^C and ^18^O/^16^O) obtained from tooth enamel. These have been used to gain insight into dietary habits and seasonal dietary variations of hominins that lived millions of years ago [6]. We used the published carbon and oxygen isotope ratios from *Paranthropus robustus* teeth and constructed power spectra using the technique applied to our hair samples. We show that the stable isotope spectra of this bipedal distant human relative gleaned from tooth enamel, who inhabited the South African savanna ∼1.8 million years ago, have well delineated slow and fast periodicities similar to those found in hair from modern humans attesting to the ANS's modulation of food and water intake and of life-style millennia ago (Fig. 4). Additionally this analysis suggests that both teeth and hair in archeological samples, as well as in modern material, are repositories of biologic rhythms that can be analyzed. Such analyses can provide clues to life style and ANS function in extant and extinct animals.

Consistent with our results is the hypothesis (Bromage TG., (2007), personal communication) which states that heart rate/pressure variability is intimately related to resource distribution to cells throughout the organism and thus to metabolic balance. The resources for metabolic activity which vary throughout the seasons are distributed by blood vessels and affected by variations in blood pressure. The rhythmic variations in resources influence stable isotope ratios in tissues resulting in the circannual variability found in hair and teeth reported here. It is conceivable, therefore, that the rhythms we observed are secondary effects of ANS control on heart rate, which in turn “feeds” that rhythm to the rest of the organism.

### d) Clinical implications

Medicine and therapeutics seek not only to cure but also to prevent irreparable damage to tissues. Biologic markers of pre-symptomatic neurodegenerative diseases will serve this goal once effective treatment and neuroprotective drugs become available. Recently it has been shown that rapid- eye-movement sleep behavior disorder (REM-BD), a parasomnia, is a biologic marker for neurodegenerations that antedates the clinical onset of these diseases by several years [13]. REM-BD is intimately linked to abnormalities in biologic rhythms in these patients and, as we show here, long hair contains a record of these rhythms spanning years. Thus, the power spectra derived from hydrogen isotope ratio in hair from individuals at risk for neurodegenerative diseases may give early warning and perhaps lead to neuroprotective strategies before irreparable nervous system damage becomes clinically apparent. Similarly, in patients with Parkinson's disease up to ∼70% of neurons in the substantia nigra will have been lost before the diagnosis is clear. Special tests have demonstrated sub-clinical alterations in ANS function early in the course of the illness [14]. Here too neuronal protective treatment may delay symptomatic impairment if the disease is recognized early enough, improving the quality of life in the terminal stages of the disease. And the subclinical ANS signs would be reflected in biologic rhythms that could be recorded from stable isotope ratios in hair.

Biologic rhythms control many aspects of physiology and behavior. The “master” timekeeper in the suprachiasmatic nucleus of the hypothalamus synchronizes numerous autonomous peripheral tissue clocks. Additionally, assigning specific functions to mammalian clock proteins that have tissue specific activity important in integrative physiology has recently been successful [15].

The hair follicle contains numerous stem cells and is the only organ that undergoes life-long cycling through growth, regression and rest periods. A “hair cycle clock” is now believed to exist, but the molecular mechanism underlying its activity remains elusive [16]. We suggest that hair specific clock proteins under the synchronizing influence of the “master time keeper” are likely to control the biologic cycling of hair, entrained in part by the ANS. The activity of these clock proteins can now be accessed through the analysis of stable isotope ratios in hair and teeth. Such analyses may have clinical applications.

## Materials and Methods

We obtained IRB approval from the Ladakh Institute of Prevention (For the study of Environmental, life-style related and High altitude Diseases) Ladakh, India and the Medicina Interna II, Ospedale L. Sacco, Università degli Studi di Milano, Milan, Italy for the examination of hair samples from living subjects. All studies were performed in accordance with the declaration of Helsinki (2002) of the World medical Association. Subjects gave written informed consent.

Hair from the mummified head of the young South American man was donated by the Department of Animal and Human Biology, the Museum of Anthropology, University of Turin, Italy. A meta-analysis of raw data from published reports was also used.

### Hair analysis

We examined hair from: a mummified child aged 7–10 years, sacrificed at 5300 m. on mount Aconcagua (Argentina) roughly 370 years ago (from reference 8); a mummified young man, radiocarbon accelerator date 1418–1491 AD (from South America); a woolly mammoth >10,000 years ago (from West Siberia) (from reference 8); the tail hair from a modern horse (USA) (from reference 8); two modern high altitude natives born and living at 4550 m in Ladakh (India), a modern young woman living in Italy and a patient with pure autonomic failure (PAF) also from Italy.

Hydrogen isotope ratios were determined using the continuous-flow-high-temperature-reduction technique [8]. Briefly, hair was wrapped in silver foil and admitted into the combustion chamber of a mass spectrometer using a Carlo Erba AS 200-LS autosampler. We sampled 5–10 mm long sections of hair beginning at the scalp or horse-tail root and 1mm long sections of hair for the mammoth and the PAF patient.

The stable isotopic compositions of low mass elements such as hydrogen are reported as “delta” (Ð) values in parts per thousand (‰). Ð values are calculated: in (‰) = (R sample/R standard-1)1000 where “R” is the ratio of the heavy to the light isotope in the sample The stable isotope standard for hydrogen is reported relative to SMOW (**S**tandard **M**ean **O**cean **W**ater). Isotope composition is reported in relation to this standard which has been defined as 0‰. [17]. Results of the hydrogen isotope ratios are given in conventional Ð notations relative to SMOW.

The series of isotope ratios derived from consecutive fragments of hair along its length or teeth growth lines were subsequently analyzed with spectral methods.

Where an annual cycle in a time series was evident, this sinusoidal cycle was fit using nonlinear regression and removed from the series. The residual series contains fast and slow periodicities (weeks/cycle) computed from the power density spectra. The annual growth rates of hair or enamel of teeth were computed from the annual sinusoid cycles.

### Analysis of teeth

We evaluated the carbon isotope and oxygen isotope ratios obtained along growth-lines of four teeth from Paranthropus robustus, a hominin that lived ∼1.8 million years ago in the South African savannah (from reference 6).

The series of isotope ratios derived from consecutive growth-lines of teeth were subsequently analyzed with spectral methods. We applied the same methodology used in the analysis of the time series of hydrogen isotope ratios in hair to the annual cycling of oxygen and carbon isotope ratios in the enamel growth lines of the hominins.

### Heart period analysis

We computed power spectra of heart rate, systolic blood pressure and respiration from the woman and the PAF patient both from Italy to compare with our spectra of the hydrogen isotope ratios of hair from the same subjects (for extensive descriptions of methodology see reference 11). Spectral indices of heart rate variability represent the dynamic interaction between the neural control of the heart and its response. The low frequencies reflect autonomic sympathetic modulation and the high frequencies autonomic parasympathetic control of heart rate variability.

The exploitation of the mean growing rates of hair and tooth enamel allowed us to derive the sampling frequency of our series and therefore, to estimate the periodicity of the observed oscillations. For methodologies see Text S1.

## Supporting Information

Text S1 Computing high and low periodicities from spectra and growth rates.
(0.09 MB DOC)
