# Supplementary material for: Stable Isotope Ratios in Hair and Teeth Reflect Biologic Rhythms
Source: PLoS One. 2007 Jul 25;2(7):e636. doi: 10.1371/journal.pone.0000636 (PMC1919425; doi:10.1371/journal.pone.0000636)
Supplement: Text S1 — Computing high and low periodicities from spectra and growth rates. (0.09 MB DOC) [file pone.0000636.s001.doc]

Text S1

# STABLE ISOTOPE RATIOS IN HAIR AND TEETH REFLECT BIOLOGIC

**RHYTHMS.**

Otto Appenzeller, Clifford Qualls, Franca Barbic, Raffaello Furlan, Alberto Porta.

Computing high and low periodicities from spectra and growth rates.

The intent here is to discuss the computation of high and low periodicities from the spectrum (periodogram) of a time series as a function of linear distance, such as, length along a strand of hair. The distance measure might also be *perikymata groove intervals* or growth lines on the surface of tooth enamel. We use annual growth rates in these measures to express high and low frequency peaks in the spectrum as periodicities in units of years (or weeks).

All spectra in the appendix were computed using the finite Fourier transform, decomposes time series into a sum of sine and cosine waves of different amplitudes and wavelengths. (PROC SPECTRA from SAS).

Determination of a high frequency peak is usually clear from the spectrum. However, determination of a low frequency peak can take several forms.

**Horse Tail Hair.** In the time series of hydrogen isotope ratio measurements (dD) every 1.5 cm along a tail hair taken from a horse [1] (from Sharp at al. . J Archeolog Sci 2003; 30:1709-1716), there is a clear annual sinusoid. The periodicity of this sinusoid is 52 weeks, but to illustrate our computations we use the growth rate of the hair to compute this periodicity. Using the span of the best fit annual sinusoid, the growth rate for the tail hair of the horse is 46 cm/year. Fitting the annual sinusoid as well as a trend by nonlinear regression yielded the function of length along the hair in cm:

Predicted dD = -.93 +.27*cm +7.08*sin (.137*cm +8.73),

as reported in Sharp et al. The frequency of the sinusoid is 0.137 radians/cm. Since by our convention all of our spectra are plotted versus frequency / $2\pi$on the interval 0 to 0.5, we calculate (0.137 radians/cm)/ ($2\pi$radians/cycle) = 0.0218 cycles/cm. Applying the inferred growth rate, our frequency is (0. 0218 cycles/cm) x (46 cm/52 weeks) = 0.0193 cycles/week and the reciprocal of frequency gives the corresponding periodicity of 52 weeks/cycle, as promised.

To summarize, when there is a clear annual sinusoid, one can use the frequency of the fitted sinusoid to estimate the growth rate that matches the 0.0193 cycles/week =

1 cycle/52 weeks, whose periodicity is 52 weeks. So (freq/$2\pi$)*(growth/year)/(52 weeks/year set equal to 1 cycle/ 52 weeks implies

growth/year = $2\pi$/(frequency of fitted annual sinusoid).

Here growth/year = $2\pi$/ .137 = 46 cm/year.

To illustrate computations from spectra, we compute the periodicity of the high frequency spectral peak in the spectrum of the horse tail hair. The horse hair time series was detrended and had the annual sinusoid removed simultaneously by nonlinear regression; the spectrum of the residual times series showed a high frequency peak at 0.263 cycles/observation on the x-axis (frequency/$2\pi$axis). The measurement interval is

dt = 1.5 cm/observation; so we calculate a frequency of (0.263 cycles/observation) /dt =

0.175 cycles/cm. Applying the growth rate, we calculate a frequency of

(0.175 cycles/cm) x (46 cm/ 52 weeks) = 0.155 cycles/week and the reciprocal gives a high frequency periodicity of 6.5 weeks/cycle.

The horse hair spectrum also showed another peak at 0.10 cycles/observation. The corresponding periodicity is

$$Period=\frac{dt}{\left(freq\right)\left(growth\right)}=\frac{1.5 cm/obs}{\left(0.10 cycles/obs\right)\left(46 cm/52 wk\right)}=17 wks/cycle.$$

One should check whether the above results might be contaminated by the folding back of high frequency content (of important power) from beyond the Nyquist folding frequency.

$$\begin{matrix} Nyquist=\pi/dt \left(radians per unit distance\right) \\ =\left(\pi/dt\right)/2\pi=0.5/dt \left(cycles per unit distance\right) . \end{matrix}$$

Note that a sparse sampling rate (large dt) yields a small Nyquist folding frequency and allows more possibility for fold-back contamination of the computed spectrum.

For the horse tail hair, Nyquist folding frequency = 0.5/1.5 = 0.333 cycle/ cm. This frequency times the growth rate gives a frequency of 0.295 cycles/week with a corresponding periodicity of 3.4 weeks. If we do not anticipate important frequencies this high or higher (periodicities this low or lower), then there should be no fold-back contamination in the above results. A more detailed discussion includes computing the aliases of our peak frequencies. The aliases of a given frequency (*f*) are

$f\pm2k Nyquist,for k=\pm1, \pm2, \pm3, \cdots$.

For discrete sampled time series, the power spectra computed at *f* is the sum of the power spectra across all aliases. For the high frequency spectral peak at 0.155 cycles/week, the relevant aliases are 0.155 ± 2x0.295 cycles/week or -0.435 and 0.745, whose corresponding periodicities are 2.3 and 1.3 weeks. Since these periodicities are unlikely to have high power, our computed high frequency peak of periodicity 6.5 weeks is unlikely to be contaminated by our sampling rate. The important aliases for the 0.067 cycles/week peak are -.523 and 0.657, whose corresponding periodicities are 1.9 and 1.5 weeks. Again our 17 week periodicity is unlike to be contaminated.

**Mammoth Hair.** In the time series of hydrogen isotope ratio measurements at multiples of 0.3 cm along a hair taken from a Mammoth [1] (Sharp et al 2003), there is a partial annual sinusoid evident. The periodicity of this sinusoid is 52 weeks, but to illustrate our computations we use the growth rate of the hair to compute this periodicity. Using the half span of the annual sinusoid, the best fit growth rate for the hair of the Mammoth is approximately 32 cm/year. Fitting the annual sinusoid as well as a trend yields the function of length along the hair in cm:

Predicted dD = -158 -.727*cm +8.69*sin (-.196*cm +3.98),

as reported in Sharp et al. The frequency of the sinusoid is 0.196 radians/cm. To convert from radians to cycles, we calculate (0.196 radians/cm)/ ($2\pi$radians/cycle) = 0.0312 cycles/cm. Applying the growth rate, our frequency is (0.0312 cycles/cm) x (32 cm/52 weeks) = 0.0192 cycles/week and the reciprocal of frequency gives the corresponding periodicity of 52 weeks/cycle.

We now compute the periodicity of the high frequency spectral peak in the spectrum of the Mammoth hair. The Mammoth hair time series was detrended and had the annual sinusoid removed simultaneously by nonlinear regression. The residual time series retains the gaps that the original series had in terms of not having measurements at every multiple of 0.3 cm, but the three different segments of data can be concatenated in the residual series to form one pooled time series whose spectrum may reveal periodicities lower than the annual one. This spectrum does showed a high frequency peak at 0.42 cycles/observation on the x-axis (frequency/$2\pi$axis). The measurement interval is

dt = 0.3 cm/observation; so we calculate a frequency of (0.42 cycles/observation) /dt =

1.4 cycles/cm. Applying the growth rate, we calculate a frequency of

(1.4 cycles/cm) x (32 cm/ 52 weeks) = 0.862 cycles/week and the reciprocal gives a high frequency periodicity of 1.2 weeks/cycle.

The Mammoth hair spectrum also showed another peak at 0.15 cycles/observation. The corresponding periodicity is

$$Period=\frac{dt}{\left(freq\right)\left(growth\right)}=\frac{0.3 cm/obs}{\left(0.15 cycles/obs\right)\left(32 cm/52 wk\right)}=3.25 wk/cycle.$$

For Mammoth hair, Nyquist folding frequency = 0.5/0.3 = 1.67 cycle/ cm. This frequency times the growth rate gives a frequency of 1.03 cycles/week with a corresponding periodicity of approximately 1.0 week. Since we do not anticipate important periodicities this low or lower, there should be no fold-back contamination in the above results.

**South America Mummies.** In the time series of hydrogen isotope ratio measurements (dD) every 0.5 cm along a hair taken from a **child** mummy [1](Sharp et al. 2003), there is a clear annual sinusoid. Fitting the annual sinusoid by nonlinear regression yielded the function of length along the hair in cm:

Predicted dD = -40.4 +21.17*sin(-.524*cm+7.52).

The frequency of the sinusoid is 0.524 radians/cm. To convert from radians to cycles, we calculate (0.524 radians/cm)/ ($2\pi$radians/cycle) = 0.083 cycles/cm. Applying the best fit hair growth rate for South American human mummies of 12 cm/year, our frequency is (0.083 cycles/cm) x (12 cm/52 weeks) = 0.0192 cycles/week and the reciprocal of frequency gives the corresponding periodicity of 52 weeks/cycle.

In the time series of hydrogen isotope ratio measurements (dD) every 1.5 cm along a hair taken from a South America **adult** mummy, there is a clear annual sinusoid of the same frequency (0.524 radians/cm) as the child. The first two time points do not match this sinusoid and are deleted. Fitting the annual sinusoid by nonlinear regression yielded the function of length along the hair in cm:

Predicted dD = -49.78 + 0.46*cm + 6.68*sin(-.524*cm + 0.983).

Since we will combine the adult data with the child data, we repeat each time point of the adult series three times so that dt = 0.5 cm to match that of the child.

We now compute the periodicity of the high frequency spectral peak in the spectrum of the combined South American human hair. The human hair time series was detrended and had the annual sinusoid removed simultaneously for the child and adult hair separately as described above. The residual time series are concatenated one pooled time series. This spectrum does showed a high frequency peak at 0.286 cycles/observation on the x-axis (frequency/$2\pi$axis). The measurement interval is dt = 0.5 cm/observation; so we calculate a frequency of (0.286 cycles/observation) /dt =0.572 cycles/cm. Applying the growth rate, we calculate a frequency of (.572 cycles/cm) x (12 cm/ 52 weeks) = 0.132 cycles/week and the reciprocal gives a high frequency periodicity of 7.6 weeks/cycle.

The South American hair spectrum also showed another peak at 0.143 cycles/observation. The corresponding periodicity is

$$Period=\frac{dt}{\left(freq\right)\left(growth\right)}=\frac{0.5cm/obs}{\left(0.143 cycles/obs\right)\left(12 cm/52 wk\right)}=15.2 wk/cycle.$$

For South American hair, Nyquist folding frequency = 0.5/0.5 = 1.0 cycle/ cm. This frequency times the growth rate gives a frequency of .23 cycles/week with a corresponding periodicity of 4.3 weeks. Since we do not anticipate important periodicities this low or lower, there should be no fold-back contamination in the above results.

**Ladakh Young adults.** In the time series of hydrogen isotope ratio measurements (dD) every 1.6 cm along a hair taken from a female, there is a clear annual sinusoid. Fitting the annual sinusoid by nonlinear regression yielded the function of length along the hair in cm:

Predicted dD = -101.8 + 7.15*sin(.352*cm – 2.778).

The frequency of the sinusoid is 0.352 radians/cm. To convert from radians to cycles, we calculate (0.352 radians/cm)/ ($2\pi$radians/cycle) = 0.056 cycles/cm. Applying the best fit hair growth rate for Ladakh human of 17.8 cm/year, our frequency is (0.056 cycles/cm) x (17.8 cm/52 weeks) = 0.0192 cycles/week and the reciprocal of frequency gives the corresponding periodicity of 52 weeks/cycle. For the **Ladakh** male, the series is shorter and there is no clear sinusoid, so we detrend this series with the predicted function;

Predicted dD = -77.5 – 1.53*cm.

We pool the residual series in order to compute a spectra for the Ladakh hair. This spectrum shows a high frequency peak at 0.379 cycles/observation on the x-axis (frequency/$2\pi$axis). The measurement interval is dt = 1.6 cm/observation; so we calculate a frequency of (0.379 cycles/observation) /dt =0.237 cycles/cm. Applying the best fit growth rate, we calculate a frequency of (.237 cycles/cm) x (17.8 cm/ 52 weeks) = 0.081 cycles/week and the reciprocal gives a high frequency periodicity of 12.3 weeks/cycle. The Ladakh hair spectrum also showed another peak at 0.207 cycles/observation. The corresponding periodicity is

$$Period=\frac{dt}{\left(freq\right)\left(growth\right)}=\frac{1.6 cm/obs}{\left(0.207 cycles/obs\right)\left(17.8 cm/52 wk\right)}=22.6 wk/cycle.$$

For Ladakh hair, Nyquist folding frequency = 0.5/1.6 = .31 cycle/ cm. This frequency times the growth rate gives a frequency of .106 cycles/week with a corresponding periodicity of 9.4 weeks. A more detailed discussion includes computing the aliases of our peak frequencies. The aliases of a given frequency (*f*) are

$f\pm2k Nyquist,for k=\pm1, \pm2, \pm3, \cdots$.

For the high frequency spectral peak at 0.081 cycles/week, the relevant aliases are 0.081 ± 2x.106cycles/week or -0.131 and 0.293, whose corresponding periodicities are 7.6 and 3.4 weeks. Our computed high frequency peak of periodicity 12.3 weeks is unlikely to be contaminated by our sampling rate. The important aliases for the 0.044 cycles/week peak are -.168 and 0.256, whose corresponding periodicities are 6.0 and 3.9 weeks. Again our 22.6 week periodicity is unlike to be contaminated.

**Young Italian woman.** In the time series of hydrogen isotope ratio measurements (dD) every 1.0 cm along a hair taken from a female, there is a clear annual sinusoid over more than two years of data. Fitting the annual sinusoid by nonlinear regression yielded the function of length along the hair in cm:

Predicted dD = -70.17 – 0.096*cm + 1.641*sin(.371*cm + .308).

The frequency of the sinusoid is 0.371 radians/cm. To convert from radians to cycles, we calculate (0.371 radians/cm)/ ($2\pi$radians/cycle) = 0.059 cycles/cm. Applying the best fit hair growth rate of 16.9cm/year, our frequency is (0.059 cycles/cm) x (16.9 cm/52 weeks) = 0.0192 cycles/week and the reciprocal of frequency gives the corresponding periodicity of 52 weeks/cycle.

We now compute the periodicity of the high frequency spectral peak in the spectrum of the combined Italian woman’s hair. The human hair time series was detrended and had the annual sinusoid removed as described above. This spectrum does showed a high frequency peak at 0.346 cycles/observation on the x-axis (frequency/$2\pi$axis). The measurement interval is dt = 1.0 cm/observation; so we calculate a frequency of (0.346 cycles/observation) /dt =0.346 cycles/cm. Applying the growth rate, we calculate a frequency of (.346 cycles/cm) x (16.9 cm/ 52 weeks) = 0.112 cycles/week and the reciprocal gives a high frequency periodicity of 8.9 weeks/cycle.

The Italian hair spectrum also showed another peak at 0.192 cycles/observation. The corresponding periodicity is

$$Period=\frac{dt}{\left(freq\right)\left(growth\right)}=\frac{1.0 cm/obs}{\left(0.192 cycles/obs\right)\left(16.9 cm/52 wk\right)}=16 wk/cycle.$$

Another peak frequency of .096 cycles/observation yields a corresponding periodicity of

$$Period=\frac{dt}{\left(freq\right)\left(growth\right)}=\frac{1.0 cm/obs}{\left(0.096 cycles/obs\right)\left(16.9 cm/52 wk\right)}=32 wk/cycle.$$

Since such low frequencies are excluded from consideration of spectra obtained from heart rate variability we marked this very low frequency by an X in fig. 2.s

For Italian hair, Nyquist folding frequency = 0.5/1.0 = .5 cycle/ cm. This frequency times the growth rate gives a frequency of .163 cycles/week with a corresponding periodicity of 6.2 weeks. The aliases of a given frequency (*f*) are

$f\pm2k Nyquist,for k=\pm1, \pm2, \pm3, \cdots$.

For the high frequency spectral peak at 0.081 cycles/week, the relevant aliases are 0.112 ± 2x.163 cycles/week or -0.213 and 0.437, whose corresponding periodicities are 4.7 and 2.3 weeks. Our computed high frequency peak of periodicity 8.9 weeks is unlikely to be contaminated by our sampling rate. The important aliases for the 0.062 cycles/week peak are -.263 and 0.387, whose corresponding periodicities are 3.8 and 2.6 weeks. Again our 16 week periodicity is unlike to be contaminated. The important aliases for the 0.031 cycles/week peak are -.294 and 0.356, whose corresponding periodicities are 3.4 and 2.8 weeks. Again our 32 week periodicity is unlike to be contaminated.

**Pure Autonomic Failure, (PAF) Older Italian Male.** In the time series of hydrogen isotope ratio measurements (dD) every 0.1 cm along a hair taken from a Italian male with diagnosed Pure Autonomic Failure , there is no clear annual sinusoid. The spectral analysis of the standardized series (mean=0, standard deviation=1) shows no spectral content.

**Perikymata – Oxygen and Carbon Ratio.** In the time series of oxygen and carbon isotope ratios (dD) per perikymata groove (pk) in **Paranthropus** teeth, there were no obvious annual sinusoids. There were four (4) teeth that were concatenated to provide a time series of adequate length (n=45 oberservations). The series was standardized within each tooth before concatenation. The growth rate used was 50 perikymata grooves per year [2] (Sponheimer et al. 2006). The spectral analyses of the standardized series (mean=0, standard deviation=1) do show spectral content.

For the **Paranthropus** **Oxygen**, there is a high frequency peak at 0.333 cycles/observation on the x-axis (frequency/$2\pi$axis). The average measurement interval is dt = 6.5 pk/observation; so we calculate a frequency of (0.333 cycles/observation) /dt =0.051 cycles/pk. Applying the growth rate, we calculate a frequency of (.051 cycles/pk) x (50 pk/ 52 weeks) = 0.049 cycles/week and the reciprocal gives a high frequency periodicity of 20 weeks/cycle. The Paranthropus Oxygen spectrum also showed another peak at 0.178 cycles/observation. The corresponding periodicity is

$$Period=\frac{dt}{\left(freq\right)\left(growth\right)}=\frac{6.5 pk/obs}{\left(0.178 cycles/obs\right)\left(50pk/52 wk\right)}=38 wk/cycle.$$

For the **Paranthropus** **Carbon**, there is a high frequency peak at 0.378 cycles/observation on the x-axis (frequency/$2\pi$axis). The average measurement interval is dt = 6.5 pk/observation; so we calculate a frequency of (0.378 cycles/observation) /dt =0.058 cycles/pk. Applying the growth rate, we calculate a frequency of (.058 cycles/pk) x (50 pk/ 52 weeks) = 0.056 cycles/week and the reciprocal gives a high frequency periodicity of 18 weeks/cycle. The Paranthropus Oxygen spectrum also showed another peak at 0.222 cycles/observation. The corresponding periodicity is

$$Period=\frac{dt}{\left(freq\right)\left(growth\right)}=\frac{6.5 pk/obs}{\left(0.222 cycles/obs\right)\left(50pk/52 wk\right)}=30 wk/cycle.$$

For Paranthropus teeth, Nyquist folding frequency = 0.5/6.5 = .077 cycle/ pk. This frequency times the growth rate gives a frequency of .074 cycles/week with a corresponding periodicity of 13.5 weeks. The aliases of a given frequency (*f*) are

$f\pm2k Nyquist,for k=\pm1, \pm2, \pm3, \cdots$.

For the Oxygen high frequency spectral peak at 0.049 cycles/week, the relevant aliases are 0.049 ± 2x0.074 cycles/week or -0.099 and 0.197, whose corresponding periodicities are 10 and 5 weeks. The important aliases for the 0.027 cycles/week peak are -.121 and 0.175, whose corresponding periodicities are 8.3 and 5.7 weeks.

For the Carbon high frequency spectral peak at 0.056 cycles/week, the relevant aliases are 0.056 ± 2x0.074 cycles/week or -0.092 and 0.204, whose corresponding periodicities are 10.9 and 4.9 weeks. The important aliases for the 0.033 cycles/week peak are -.115 and 0.181, whose corresponding periodicities are 8.7 and 5.5 weeks.

Reference List.

1. Sharp,Z.D. Atudorei,V. Panarello,H.O. Fernández,J. Douthitt,C.

Hydrogen isotope systematics of hair: archeological and forensic applications. J Archeolog Sci 2003; 30:1709-1716.

2. Sponheimer,M. Passey,B.H. de Ruiter,D.J. Guatelli-Steinberg,D. Cerling,T.E. et al.

Isotopic evidence for dietary variability in the early hominin *Paranthropus robustus*. Science 2006; 314:980-981.
